# Efficacy and safety of methylene blue in patients with vasodilatory shock: A systematic review and meta-analysis

**DOI:** 10.3389/fmed.2022.950596

**Published:** 2022-09-26

**Authors:** Cong-Cong Zhao, Yu-Jia Zhai, Zhen-Jie Hu, Yan Huo, Zhi-Qiang Li, Gui-Jun Zhu

**Affiliations:** ^1^Department of Intensive Care Unit, The Fourth Hospital of Hebei Medical University, Shijiazhuang, China; ^2^Department of Intensive Care Unit, Affiliated Hospital of North China University of Science and Technology, Tangshan, China

**Keywords:** methylene blue, vasodilatory shock, vasoplegic syndrome, septic shock, mortality, meta-analysis

## Abstract

**Background:**

The role of methylene blue (MB) in patients with vasodilatory shock is unclear. The purpose of this systematic review and meta-analysis was to evaluate the efficacy and safety of MB in patients with vasodilatory shock.

**Methods:**

We searched MEDLINE at PubMed, Embase, Web of Science, Cochrane, CNKI, CBM and Wanfang Medical databases for all observational and intervention studies comparing the effect of MB vs. control in vasodilatory shock patients. This study was performed in accordance with the PRISMA statement. There were no language restrictions for inclusion.

**Results:**

A total of 15 studies with 832 patients were included. Pooled data demonstrated that administration of MB along with vasopressors significantly reduced mortality [odds ratio (OR) 0.54, 95% confidence interval (CI) 0.34 to 0.85, *P* = 0.008; *I*^2^ = 7%]. This benefit in mortality rate was also seen in a subgroup analysis including randomized controlled trials and quasi-randomized controlled trials. In addition, the vasopressor requirement was reduced in the MB group [mean difference (MD) −0.77, 95%CI −1.26 to −0.28, *P* = 0.002; *I*^2^ = 80%]. Regarding hemodynamics, MB increased the mean arterial pressure, heart rate and peripheral vascular resistance. In respect to organ function, MB was associated with a lower incidence of renal failure, while in regards to oxygen metabolism, it was linked to reduced lactate levels. MB had no effect on the other outcomes and no serious side effects.

**Conclusions:**

Concomitant administration of MB and vasopressors improved hemodynamics, decreased vasopressor requirements, reduced lactate levels, and improved survival in patients with vasodilatory shock. However, further studies are required to confirm these findings.

**Systematic review registration:**

Identifier: CRD42021281847.

## Introduction

Vasodilatory shock is defined as life-threatening acute circulatory failure characterized by low arterial pressure, normal or elevated cardiac output, and reduced systemic vascular resistance, resulting in inadequate oxygen utilization (1, 2). It can be related to various causes (i.e., sepsis, vasoplegic syndrome, liver transplant, and allergy) and the final stage of other types of shock. Treatment is centered upon providing adequate organ reperfusion and oxygen utilization by fluid resuscitation and catecholamine vasopressors. However, high doses of catecholamines increase the risk of adverse effects such as peripheral ischemia/dysfunction, tachyarrhythmia, myocardial depression, and others (3, 4). A recent study reported that excessive doses of norepinephrine was associated with acute kidney injury (AKI) and intensive care unit (ICU) mortality following cardiac surgery (5). Moreover, first-line norepinephrine is inefficacious in some patients (6), therefore, researchers are actively looking for catecholamine-sparing agents.

Methylene blue (MB), a water-soluble dye and an inhibitor of nitric oxide (NO), is an alternative method to restore vascular tone and improve perfusion (7). In vasodilatory shock, elevated levels of NO and activation of soluble guanylyl cyclase (sGC) are the main reasons for the mismatch between macrocirculation and microcirculation (8). MB inhibits NO and selectively inhibits inducible NO synthase generation. Additionally, MB binds to the heme part of sGC, blocking the effect of sGC in vascular smooth muscle, reducing the level of cyclic adenosine monophosphate, and synergistically improving vasodilation (9). Studies have reported that MB was able to significantly increase the mean arterial pressure (MAP) and systemic vascular resistance (SVR) with no apparent major side effect (10, 11). In addition, MB administration was able to facilitate the weaning of catecholamine vasopressors (12–14). Taken together, MB represents an option for catecholamine-sparing agents.

Although MB may improve vasodilation, a corresponding mortality benefit was not seen overall. A recent retrospective study found that MB responders had a lower mortality compared to MB non-responders (15). Li et al. reported that compared with norepinephrine monotherapy, MB combination therapy improved mortality in sepsis patients (16). However, the result could not be reproduced in subsequent studies (17, 18). Due to the lack of randomized controlled trials (RCTs) and divergent patient subsets, the efficacy of MB on mortality is unclear. Moreover, there is no consensus on several key issues, including MB treatment time window, optimal dose and administration mode. Some studies used MB as a last rescue therapeutic in refractory vasodilatory shock (15, 18, 19). This may limit the effectiveness of MB due to the treatment time later than the “window of opportunity” (20). The mode and dose of MB administration were inconsistent among all studies, which ranged from 0.5 mg/kg/h to 4 mg/kg/h by intravenous injection with or without continuous infusion (6). Thus, the role of MB in patients with vasodilatory shock remains unclear.

Therefore, we performed a meta-analysis to evaluate the efficacy and safety of MB in vasodilatory shock patients. Subgroup analyses were performed to explore the benefits of MB for different populations, modes, and administration doses.

## Methods

### Study registration

This systematic review was registered at PROSPERO with the registration number CRD42021281847. It was performed according to the Preferred Reporting Items for Systematic Reviews and Meta-Analyses (PRISMA) guidelines (21).

### Data sources

We searched the MEDLINE via the PubMed, Embase, Web of Science, Cochrane, CBM, CNKI, and Wanfang databases up to April 10, 2022, using the key words (“Methylene blue”) AND (“Shock” or “Septic” or “Vasoplegia” or “Hypotension”). There were no language restrictions. The search strategy in provided in more detail in the Supplementary material.

### Study selection

The initial and full-text reviews were performed independently by two authors (CCZ and YJZ). The inclusion criteria were as follows: 1) type of study: observational study or interventional study (either randomized or non-randomized); 2) population: adult patients (≥18 years) suffering from vasodilatory shock treated with routine fluid and catecholamine vasopressor therapy; 3) intervention: intravenous MB treatment vs. placebo or blank; 4) outcomes: the primary outcome was mortality without time limits, and secondary outcomes were vasopressor requirement; hemodynamic changes [including mean arterial pressure (MAP); systemic vascular resistance (SVR); heart rate (HR); and cardiac index (CI)]; oxygen metabolism [including lactate, oxygen delivery (DO_2_I), oxygen consumption (VO_2_I)]; organ function; intensive care unit (ICU) and hospital length of stay (LOS); mechanical ventilation duration; as well as adverse effects.

The exclusion criteria were as follows: 1) oral administration of MB or MB as a prophylactic treatment; 2) lack of a baseline condition or control group; 3) lack of data on any outcome; and 4) review articles, cohort studies, case reports and studies without full text, animal and *in vitro* studies.

### Data extraction

Two authors (CCZ and YJZ) independently extracted data from the included studies. The kappa coefficient was calculated as a measure of agreement about study selection and quality appraisal. Any discrepancies were resolved by the third author (ZQL), and a decision was reached by consensus.

For each study, the following information was extracted: publication (last name of the first author, year of publication), participant characteristics (including patient source, diagnosis, demographic data, clinical setting, and number of patients), details of the intervention (including MB dosage, route, and duration), follow-up duration, and outcome data.

### Assessment of risk of bias

Two authors independently assessed the risk of bias to evaluate the quality of the included studies. The Cochrane Collaboration tool (22) was used for RCTs, and the Newcastle–Ottawa Scale (NOS) (23) was used for non-RCTs and observational studies. A funnel plot was used to evaluate publication bias.

### Statistical analysis

SPSS 25.0 (IBM, Armonk, New York, USA) was used to calculate the kappa coefficient. Data analysis was conducted by RevMan 5.4 (The Nordic Cochrane Center, Rigs Hospitalet, Copenhagen, Denmark). The results are presented with forest plots using odds ratio (OR) for dichotomous data and the mean differences (MD) for continuous data. If continuous data with different units, the standardized mean differences (SMD) was used. All estimates were provided with 95% confidence interval (CI). Heterogeneity was assessed by Cochran's Q statistic and the *I*^2^-test. A *P* value > 0.1 or I^2^ statistic below 50% indicated low levels of heterogeneity. In these cases, a fixed-effect model was used. Otherwise, a random-effects model (Mantel–Haenszel method) was selected. *P* < 0.05 indicated statistical significance. Several subgroup analyses were performed for mortality according to population (septic shock and non-septic shock), mode (intravenous injection and continuous infusion) and the design of the trial (RCTs and non-RCTs).

## Results

### Study screening

**Figure 1:**
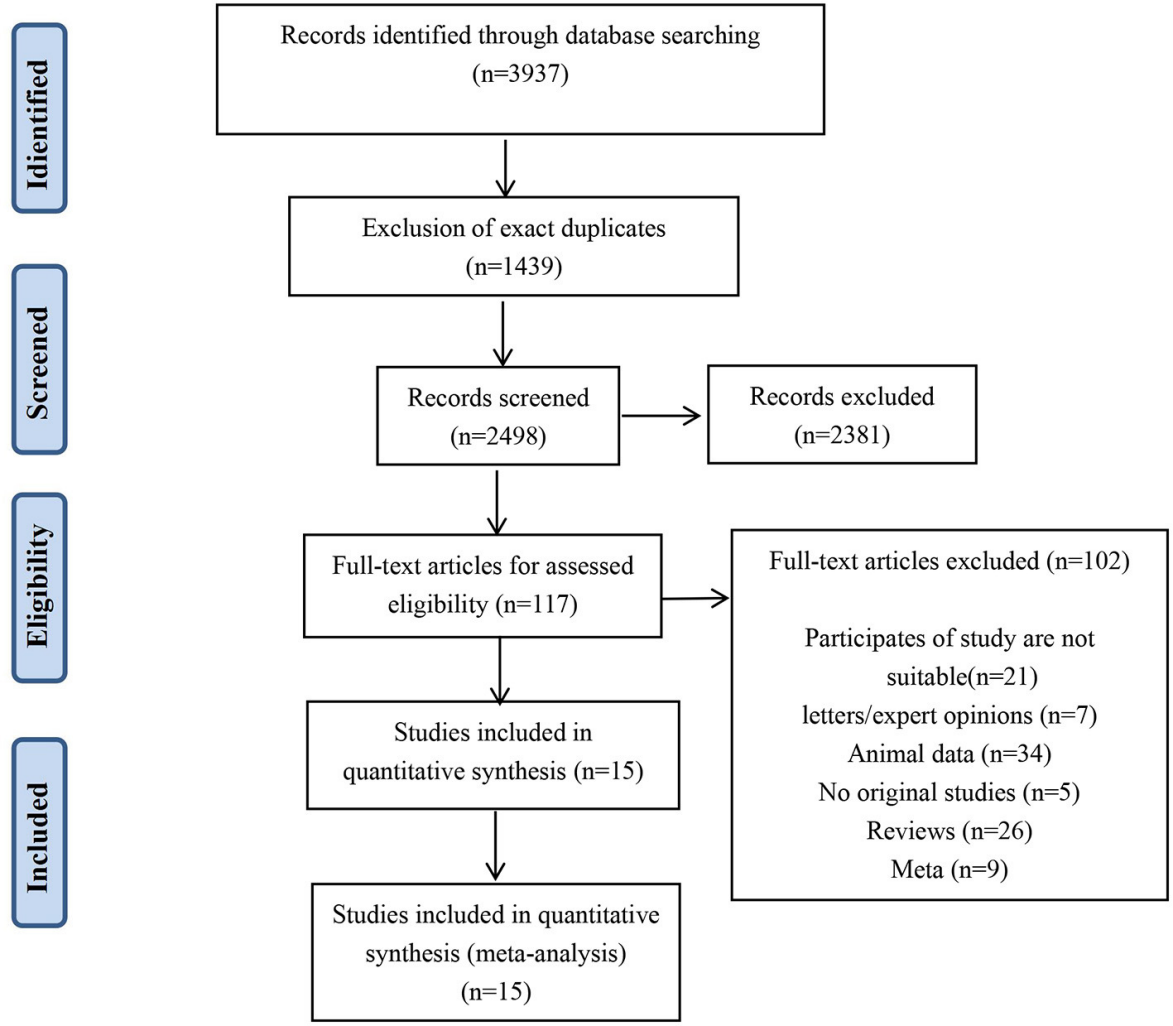
Study selection flow diagram according to the PRISMA guidelines.

The search strategy identified 3,937 unique publications. After excluding 1,439 duplicates and screening 2,381 titles and abstracts, 117 studies were assessed in full text for eligibility. The full-text screening excluded 102 studies for the reasons shown in Figure 1. Finally, 15 studies (12, 16, 23–35) were included in this meta-analysis. Among these, 7 studies were published in Chinese, and the other 8 studies were published in English.

**Figure 2:**
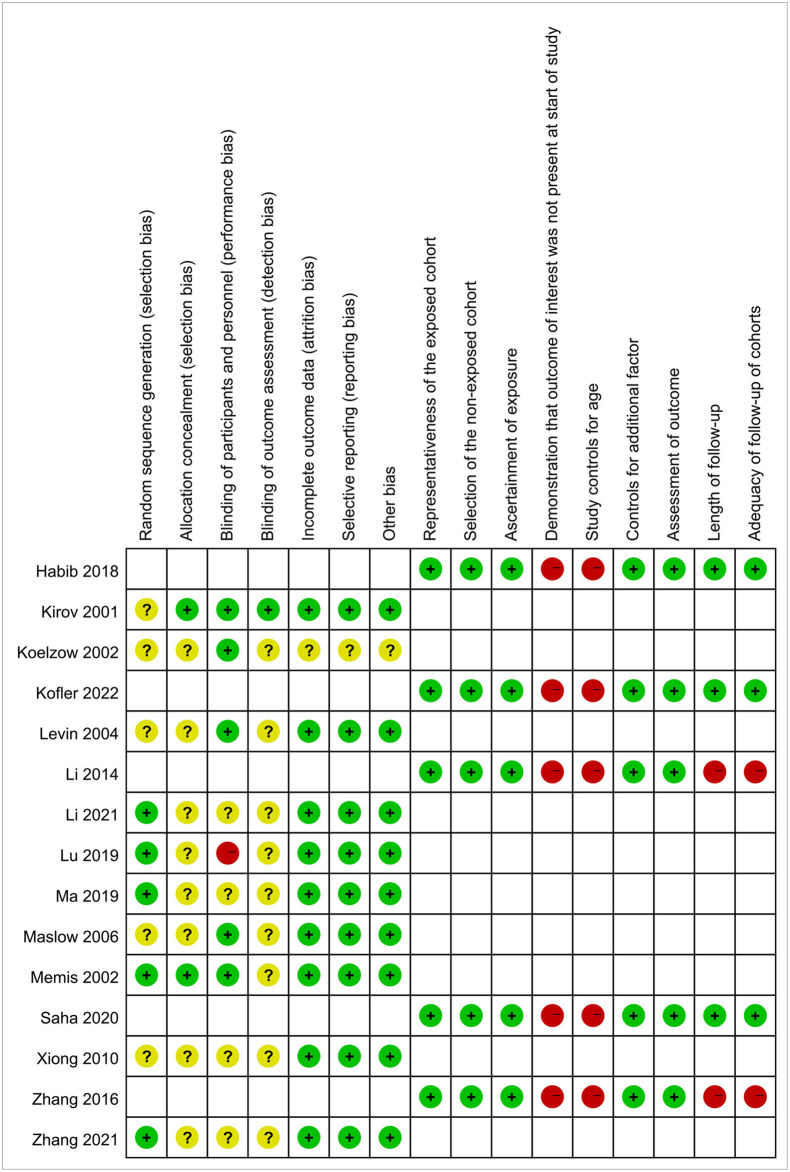
Risk of bias summary assessments for included studies.

The characteristics of all the included studies are summarized in Table 1. In total, the included studies comprised 832 patients, and the number of patients per study was 20 to 120. The population included septic shock, vasoplegic syndrome, and ischemia reperfusion. Of the 15 included studies, 7 were RCTs, 3 were quasi-Randomized Controlled Trials (q-RCTs), and 5 were observational studies. Among the 10 interventional studies, only 1 study had a high risk of bias, and all the other 9 studies had a mild to moderate risk of bias. All 5 observational studies except for 2 had a mild risk of bias. The risk bias of the included studies is shown in Figure 2.

**Table 1:** Characteristics of included studies.

**Study**	**Country**	**Design**	**Setting**	**Participants**	**MB intervention**	**Control**	**Outcomes**
Kirov et al. (12)	Russia	RCT	ICU	Septic shock *n =* 20	**Time:** after diagnosis **Dose:** 2 mg/kg (iv) +0.25–2.00 mg/kg/h (iv 4 h)	Placebo	1, 2, 3, 4, 5, 6, 8, 9, 11,12,13, 14, 15,16
Koelzow et al. (31)	UK	RCT	Surgery	Ischemia reperfusion syndrome *n =* 38	**Time:** 1 min before graft reperfusion **Dose:** 1.5 mg/kg (iv)	Placebo	1, 2, 5, 6, 7,11
Memis et al. (24)	Turkey	RCT	Anesthesiology	Septic shock *n =* 30	**Time:** after diagnosis **Dose:** 0.5 mg/kg/h (iv 6 h)	Placebo	1, 2, 3,11,12,13, 15,16
Levin et al. (33)	Argentina French Swiss	RCT	Surgery	Vasoplegic syndrome *n =* 56	**Time:** after diagnosis **Dose:** 1.5 mg/kg/h (iv 1h)	Placebo	1,10,16
Maslow et al. (32)	USA	RCT	Surgery	Vasoplegic syndrome *n =* 30	**Time:** after the onset of cardiopulmonary bypass **Dose:** 3 mg/kg (iv)	Placebo	2, 3, 4, 6, 7, 16
Habib et al. (25)	Egypt	Observational study	CSICU	Vasoplegic syndrome *n =* 56	**Time:** after diagnosis **Dose:** 2 mg/kg (iv) +0.5–1 mg/kg/h (iv, if needed)	Blank	1, 6, 10, 13, 14, 15
Saha et al. (26)	USA	Observational study	ICU	Vasoplegic syndrome *n =* 68	**Time:** after diagnosis **Dose:** 1–2 mg/kg (iv) +1–2 mg/kg (iv, if >1h)	Blank	1, 13, 14
Kofler et al. (35)	USA	Observational study	Anesthesiology	Vasoplegic syndrome *n =* 120	**Time:** after diagnosis **Dose:** 2 mg/kg (iv)	Blank	1, 2, 6, 11,12,13, 14, 15
Xiong et al. (27)	China	RCT	Anesthesiology	Septic shock *n =* 40	**Time:** after diagnosis **Dose:** 0.5–1.0 mg/kg/h (iv)	Blank	2, 3, 4, 5, 7, 8, 9
Li (36)	China	Observational study	ICU	Septic shock *n =* 86	**Time:** after diagnosis **Dose:** 4 mg/kg (iv)	Blank	2, 4, 5
Zhang and Wu (28)	China	Observational study	ICU	Septic shock *n =* 90	**Time:** after diagnosis **Dose:** 2 mg/kg (iv) + 0.25–2 mg/kg/h (iv 24h)	Blank	3, 5
Lu et al. (29)	China	RCT	ICU	Septic shock *n =* 54	**Time:** after diagnosis **Dose:** 2 mg/kg (iv) /+ 2 mg/kg/h (iv 24 h)	Placebo	1, 3, 6, 7
Ma et al. (34)	China	RCT	Anesthesiology	Vasoplegic syndrome *n =* 28	**Time:** 10 min before CPB shutdown **Dose:** 2 mg/kg (iv)	Placebo	2, 3, 7, 13, 15
Li (16)	China	q-RCT	ICU	Septic shock *n =* 66	**Time:** after diagnosis **Dose:** 2 mg/kg (iv)	Blank	1, 3, 5, 13
Zhang et al. (30)	China	RCT	Surgery	Vasoplegic syndrome *n =* 50	**Time:** after diagnosis **Dose**: 2 mg/kg (iv 3–4 h)	Blank	2, 3, 4, 8, 9

RCT, Randomized controlled trial; q-RCT, quasi-Randomized controlled trial; ICU, Intensive care unit; CSICU, Cardiac surgical intensive care unit; MB, Methylene blue; CPB, Cardiopulmonary bypass; iv, intravenous injection; Outcomes: 1 Mortality; 2 Mean arterial pressure; 3 Heart rate; 4 Systemic vascular resistance; 5 Cardiac index; 6 Vasopressor Requirement; 7 Lactate; 8, Oxygen delivery; 9, Oxygen consumption; 10, Renal failure; 11, Creatinine; 12, Alanine aminotransferase; 13 Intensive care unit length of stay; 14 Hospital length of stay; 15 Duration of Mechanical Ventilation; 16 Adverse Reaction.

### Mortality

**Figure 3:**
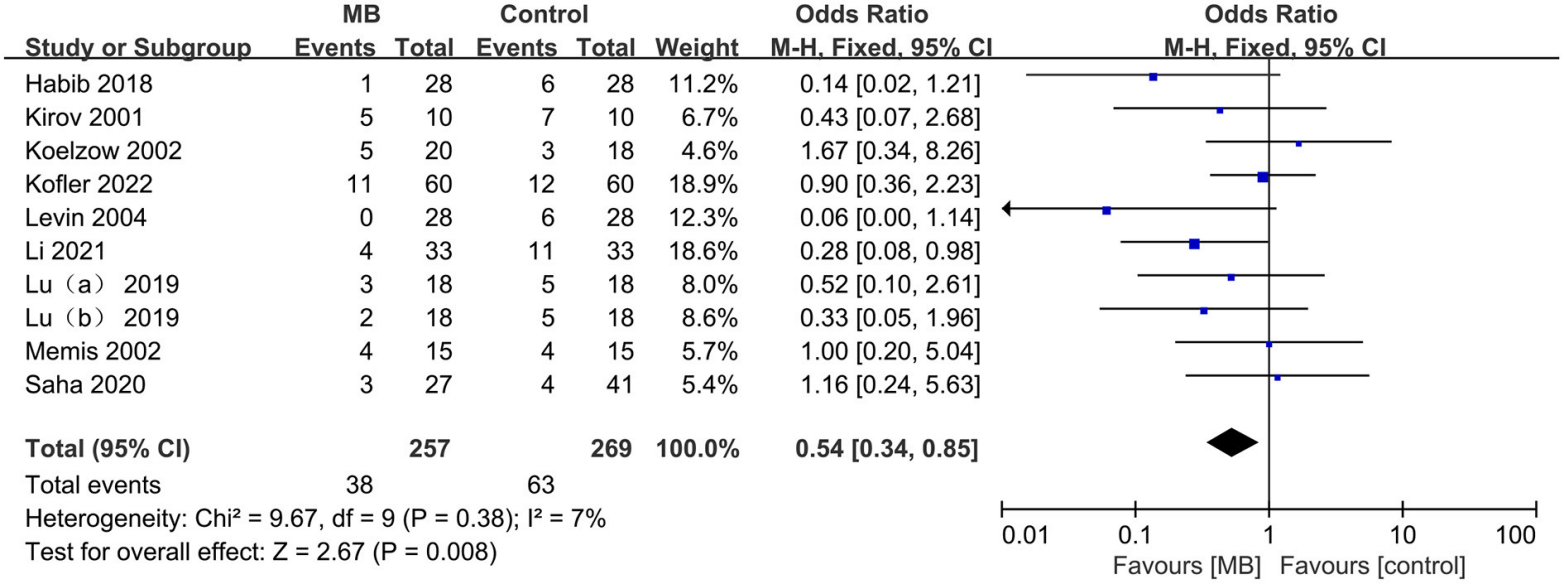
Pooled mortality regarding the longest available time period within each study, odds ratio, methylene blue treatment vs. control; M-H, Mantel–Haenszel; CI, Confidence interval.

Nine (*n* = 526, 257 in the MB group and 269 in the control group) of the 15 included studies reported mortality ranging from 0–70% with different follow-up times, including 28 days, 30 days, 90 days and hospitalization. The pooled data showed that compared with the control group, MB significantly reduced mortality in patients with vasodilatory shock (OR 0.54, 95% CI 0.34 to 0.85, *P* = 0.008; Figure 3), with low heterogeneity (*I*^2^ = 7%). No sign of significant publication bias was observed (Supplementary Figure S1).

This result was confirmed by the pooled analysis from RCTs and q-RCTs (OR = 0.45, 95%CI 0.25 to 0.81, *P* = 0.008; *I*^2^ = 1%; supplementary Figure S2), rather than non-RCTs (OR 0.70, 95%CI 0.34 to 1.42, *P* = 0.32; *I*^2^ = 29%; Supplementary Figure S2). Subgroup analyses of the population revealed a reduction in mortality in patients with septic shock (OR 0.43, 95%CI 0.22 to 0.87, *P* = 0.02; *I*^2^ = 0%; Supplementary Figure S3), but the difference was not statistically significant in non-septic shock patients (OR 0.63,95%CI 0.35 to 1.16, *P* = 0.14; *I*^2^ = 42%; Supplementary Figure S3). Continuous infusion of MB significantly improved survival (OR 0.36, 95% CI 0.15 to 0.88, *P* = 0.02; *I*^2^ = 0%; Supplementary Figure S4), while no significant difference was found between the intervention injection MB and control groups (OR 0.62, 95% CI 0.37 to 1.07, *P* = 0.08; *I*^2^ = 19%; Supplementary Figure S4). The dosages of MB used in the included studies that reported mortality were relatively uniform, ranging from 1–2 mg/kg for intravenous injection and 0.25–2 mg/kg/h for continuous infusion. Therefore, we did not perform subgroup analyses based on the doses of MB.

### Secondary outcomes

#### Vasopressor requirement

Four of the 15 included studies used MB in addition to vasopressors, such as norepinephrine, epinephrine, dopamine, and dobutamine. Four studies with 430 patients reported that MB significantly reduced the requirement for vasopressors compared with the control group (SMD −0.77, 95% CI −1.26 to −0.28, *P* = 0.002; *I*^2^ = 80%; Table 2).

**Table 2:** Secondary results of this meta-analysis.

**Covariate**	**Studies**	**MD/SMD/OR**	**LCI**	**UCI**	***P***	**I^2^**
Vasopressor requirement	4	−0.77	−1.26	−0.28	**0.002**	80
MAP	9	4.76	2.99	6.54	**<0.001**	33
HR	8	4.70	2.38	7.02	**<0.001**	71
SVR	5	181.87	39.30	324.44	**0.01**	88
CI	6	0.36	−0.03	0.74	0.07	95
Lactate	5	−0.97	−1.34	−0.59	**<0.001**	72
DO_2_I	3	−19.63	−106.30	67.04	0.66	98
VO_2_I	3	10.85	−0.13	21.84	0.05	63
Renal failure	2	0.14	0.03	0.58	**0.007**	0
Creatinine	3	0.37	−0.84	0.57	0.55	90
Alanine minotransferase	3	−0.60	−0.92	−0.28	**<0.001**	19
ICU LOS	6	−0.41	−0.99	0.17	0.16	83
Hospital LOS	4	−0.30	−9.82	9.23	0.95	87
Mechanical ventilation duration	5	−0.47	−1.06	0.13	0.13	78

P < 0.05 represented in bold OR, Odds ratio; MD, Mean deviation; SMD, standardized mean difference; LCI, Lower 95% confidence interval; UCI, Upper 95% confidence interval; MAP, Mean arterial pressure; HR, Heart rate; SVR, Systemic vascular resistance; CI, Cardiac index; DO2I, Oxygen delivery; VO2I, Oxygen consumption; ICU, Intensive care unit; LOS, Length of stay.

#### Hemodynamic changes

Nine (*n* = 440) of the 15 included studies reported MAP, which was significantly increased by MB (MD 4.76, 95% CI 2.99 to 6.54, *P* < 0.001; *I*^2^ = 33%; Table 2). Pooled data from 8 studies (*n* = 396) revealed that MB significantly increased HR (MD 4.70, 95% CI 2.38 to 7.02, *P* < 0.001; *I*^2^ = 71%; Table 2). Moreover, SVR (5 studies, *n* = 226) was also higher in the MB group than in the control group (MD 181.87, 95% CI 39.30 to 324.44, *P* = 0.01; *I*^2^ = 88%; Table 2). However, comprehensive data from 6 studies (*n* = 340) revealed no significant difference in cardiac index between the two groups (MD 0.36, 95% CI −0.03 to 0.74, *P* = 0.07; *I*^2^ = 95%; Table 2).

#### Oxygen metabolism

Five (*n* = 208) of the 15 included studies reported lactate values. The results showed that MB could significantly reduce the level of lactate (MD −0.97, 95% CI −1.34 to −0.59, *P* < 0.001; I^2^ = 72%; Table 2). However, only 3 studies reported DO_2_I or VO_2_I, and neither of them was significantly different between MB and the control groups (DO_2_I: MD −19.63, 95% CI −106.30 to 67.04, *P* = 0.66; I^2^ = 98%; VO_2_I: MD 10.85, 95% CI −0.13 to 21.84, *P* = 0.05; I^2^ = 63%; Table 2).

#### Organ function

Among the 15 included studies, two studies (*n* = 112) reported the incidence of renal failure, and three studies (*n* = 163) reported the effect of MB on creatinine. The pooled data showed that MB was associated with a lower incidence of renal failure (OR = 0.14, 95%CI 0.03 to 0.58, *P* = 0.007; I^2^ = 0%; Table 2), but had no effect on the level of creatinine (SMD 0.37, 95% CI −0.84 to 0.57, *P* = 0.55; I^2^ = 90%; Table 2). Moreover, three studies (*n* = 168) reported the data of alanine aminotransferase. The pooled data showed that MB could significantly reduce the level of alanine aminotransferase (SMD −0.60, 95% CI −0.92 to −0.28, *P* < 0.001; *I*^2^ = 19%; Table 2).

#### Other secondary outcomes

The effects of MB treatment on ICU LOS (6 studies, *n* = 332) and hospital LOS (4 studies, *n* = 264) were −0.41 days (95% CI −0.99 to 0.17, *P* = 0.16; I^2^ = 83%; Table 2) and −0.30 days (95% CI −9.82 to 9.23, *P* = 0.95; I^2^ = 87%; Table 2), respectively. Five studies (*n* = 254) reported the mechanical ventilation duration. Compared with the control group, MB had no effect on the duration of mechanical ventilation (SMD −0.47, 95% CI −1.06 to 0.13, *P* = 0.13; I^2^ = 78%; Table 2).

### Adverse effects

No serious side effects were found in this study. The adverse effects of MB reported in the included studies were blue discoloration of the skin and urine and a temporary decrease in mixed venous oxygen saturation.

## Discussion

This systematic review and meta-analysis examined the effect of MB in 15 studies including more than 800 vasodilatory shock patients with various causes, including septic shock, vasoplegic syndrome, and ischemia reperfusion injury. The main finding is that MB as a catecholamine-sparing agent may improve the survival of patients with vasodilatory shock. For secondary outcomes, MB significantly decreased the requirement for vasopressors. MB also had the beneficial effect on hemodynamic changes, organ function, and lactate level. However, MB had no effect on mechanical ventilation duration, ICU LOS or hospital LOS.

In vasodilatory shock, elevated levels of NO and activation of sGC are the main reasons for vasodilation. As an NO inhibitor, MB has the ability to restore vascular tone and increase blood pressure (6). Although MB represents another option of catecholamine-sparing agents, its role in patients with vasodilatory shock is still inconsistent due to insufficient evidence.

Our review found that compared with placebo, MB treatment significantly reduced the mortality of patients with vasodilatory shock. Consistently, Levin et al. reported that MB was associated with a lower mortality and potentially faster reversal of vasoplegia compared to placebo in vasoplegic patients (33). A recent meta-analysis by Perdhana et al. reported that compared with placebo and hydroxocobalamin, administration of MB significantly reduced mortality for vasoplegic syndrome in cardiopulmonary bypass surgery patients (37).

In contrast to our study, Furnish et al. (18) showed that as a rescue therapy for vasoplegic syndrome, there was no significant difference in mortality between the MB and hydroxocobalamin groups. The meta-analysis by Pasin et al. (38) included 5 studies with a total of 174 hypotensive patients and indicated that MB showed no detrimental effect on survival. The inconsistent effects of MB on mortality may be attributable to some potential confounding factors, including different patients, methods and dosages of MB administration. Therefore, subgroup analyses were performed. We identified a significant difference between groups favoring continuous infusion MB with a dosage of 0.25–2 mg/kg/h in septic shock patients. This result can be attributed to several reasons. First, a possible “window of opportunity” (the first 8 h) for MB's effectiveness in sepsis has been proposed (39), which indicated that MB was less effective as a late rescue therapy (20). In most of the included studies, MB was used in the early stage of vasodilatory shock patients (Table 1). Second, MB acts rapidly after intravenous injection, with a terminal plasma half-life of 5–6 h (7). Considering the short-acting effects of MB, continuous infusion for a longer time may be more effective. Third, Juffferman et al. (40) found that the infusion of 1–3 mg/kg MB could improve circulation without increasing the gastric mucosa-arterial carbon dioxide partial pressure difference. Although the high dose of methylene blue (7 mg/kg) will further increase the systemic blood flow, splanchnic blood perfusion may be compromised. In summary, our study suggests that MB use in early septic shock may benefit patients more, that continuous infusion is preferred, and that it starts with low effective doses.

Our study further analyzed the possible mechanisms by which MB improved mortality. First, it is worth noting that almost all of the included studies used MB as an adjunct intervention to catecholamine vasopressor, which is the first choice for the treatment of vasodilatory shock (41). Although catecholamine vasopressors increase blood pressure and cardiac output, high doses may be responsible for several complications, such as peripheral ischemia, dysrhythmias, and increased myocardial oxygen consumption, all of which were associated with an increased risk of death (42). This study showed that compared with the control group, the vasopressor requirement in the MB group was significantly reduced. Second, NO leads to severe hypoxia and organ failure by its direct cytotoxicity and mediated hypotension (43). MB, as a NO inhibitor, improved hemodynamics, including elevated MAP, HR, and SVR. Third, MB reduced organ failure. The pooled data showed that MB was associated with a lower incidence of renal failure, also reduced the level of alanine aminotransferase. Fourth, vasoplegia for more than 36–48 h is associated with a higher risk of multiple organ failure and death (44). Compared to conventional therapy, MB administration reduced the duration of vasoplegia by 3 times (33). As a result, the survival rate was improved. In summary, hemodynamic restoration is a crucial determinant in survival probability. The consequence of classic stepwise vasopressor approach is excessive catecholamine administration which leads to several complications and poor outcome. An early multimodal vasopressor therapy may be a better choice (45).

In addition, our study found that MB had a beneficial effect on oxygen metabolism, manifested as a decrease in lactate. Consistently, a recent meta-analysis (46) reported that serum lactate was significantly decreased after MB administration in patients with refractory hypotension. Lactate can reflect tissue oxygen metabolism and microcirculation perfusion, and its increase is closely related to high mortality (47). However, there was no difference in oxygen delivery or oxygen consumption. Considering the limited number of included studies, small sample size, and relatively high heterogeneity of this result, the effect of MB on oxygen metabolism needs to be further verified by more studies.

No serious side effects were found in the included studies in this meta-analysis. The main adverse effect of MB was blue discoloration of the skin and urine. It should be noted that MB has been found to lead to local skin necrosis, increased pulmonary vascular resistance, arrhythmias, and decreased oxygen saturation (48). Moreover, Martino et al. reported 3 cases of life-threatening serotonin toxicity, including coma, in patients who undergoing chronic selective serotonin reuptake inhibitor therapy and received MB for vasoplegic syndrome (49). Most side effects are dose-related, and the application of MB is relatively safe when the dose does not exceed 2 mg/kg (50).

To our knowledge, the present study is the most extensive systematic review and meta-analysis on the role of MB in patients with vasodilatory shock, with a broad search strategy, inclusion of extensive studies and the latest research with high methodological quality. Moreover, we performed various subgroup analyses for mortality, the main outcome of this study, and generated new hypotheses for practical applications. In addition, this study was the first to analyze the effect of MB on oxygen metabolism.

Several limitations should be considered when interpreting the findings of this study. First, many included studies were observational studies. The evidence level was not high enough. However, a subgroup analysis for RCTs was also performed in this meta-analysis. Second, the etiology of shock, severity of illness and MB intervention were diverse in the included studies. These can be a risk of bias and weaken the strength of the evidence. Third, although the number of included studies was large, all of them were restricted to small sample sizes. Therefore, large-scale clinical trials are needed to clarify the findings of this study.

## Conclusion

In conclusion, this meta-analysis suggests that the adjunction of continuous administration of MB to vasopressors may be associated with lower mortality in patients with vasodilatory shock with no severe side effects. Further large-scale RCTs are required to ascertain MB efficacy and safety.

## Data availability statement

The original contributions presented in the study are included in the article/Supplementary materials, further inquiries can be directed to the corresponding author/s.

## Author contributions

The study was designed by G-JZ. C-CZ and Y-JZ acquired the data, performed the analysis, and wrote the manuscript. Z-QL helped with the search criteria. Z-JH corrected and contributed to the manuscript. Tables were produced by YH. All authors read and approved the final manuscript.

## Conflict of interest

The authors declare that the research was conducted in the absence of any commercial or financial relationships that could be construed as a potential conflict of interest.

## Publisher's note

All claims expressed in this article are solely those of the authors and do not necessarily represent those of their affiliated organizations, or those of the publisher, the editors and the reviewers. Any product that may be evaluated in this article, or claim that may be made by its manufacturer, is not guaranteed or endorsed by the publisher.

## Abbreviations

MB: Methylene blue
OR: Odds ratio
CI: Confidence interval
M-H: Mantel–Haenszel
MD: Mean difference
SMD: Standardized mean difference
NO: Nitric oxide
sGC: soluble Guanylyl cyclase
AKI: Aacute Kidney Injury
MAP: Mean arterial pressure
SVR: Systemic vascular resistance
HR: Heart rate
CI: Cardiac index
DO_2_I: Oxygen delivery index
VO_2_I: Oxygen consumption index
RCTs: Randomized controlled trials
q-RCTs: quasi-Randomized controlled trials
NOS: Newcastle-Ottawa quality assessment scale
ICU: Intensive care unit
LOS: Length of stay.

## References

[1] Angus DC van der Poll T. Severe sepsis and septic shock. N Engl J Med. (2013) 369:2063. 10.1056/NEJMc1312359 24256390

[2] Gomes WJ Carvalho AC Palma JH Buffolo E. Vasoplegic syndrome: a new dilemma. J Thorac Cardiovasc Surg. (1994) 107:942–3. 10.1016/S0022-5223(94)70355-8 8127127

[3] Schmittinger CA Torgersen C Luckner G Dünser MW. Adverse cardiac events during catecholamine vasopressor therapy: a prospective observational study. Intens Care Med. (2012) 38:950–8. 10.1007/s00134-012-2531-2 22527060

[4] Dunser MW Hasibeder WR. Sympathetic overstimulation during critical illness: adverse effects of adrenergic stress. J Intensive Care Med. (2009) 24:293. 10.1177/0885066609340519 19703817

[5] Huette P Moussa MD Beyls C Guinot P Guilbart M Besserve P . Association between acute kidney injury and norepinephrine use following cardiac surgery: a retrospective propensity score-weighted analysis. Ann Intensive Care. (2022) 12:61. 10.1186/s13613-022-01037-1 35781575 PMC9250911

[6] Scheeren T Bakker J De Backer D Annane D Asfar P Boerma EC . Current use of vasopressors in septic shock. Ann Intensive Care. (2019) 9:20. 10.1186/s13613-019-0498-7 30701448 PMC6353977

[7] Tchen S Sullivan JB. Clinical utility of midodrine and methylene blue as catecholamine-sparing agents in intensive care unit patients with shock. J Crit Care. (2020) 57:148–56. 10.1016/j.jcrc.2020.02.011 32145658

[8] Puntillo F Giglio M Pasqualucci A Brienza N Paladini A Varrassi G. Vasopressor-sparing action of methylene blue in severe sepsis and shock: a narrative review. Adv Ther. (2020) 37:3692–706. 10.1007/s12325-020-01422-x 32705530 PMC7444404

[9] Jang DH Nelson LS Hoffman RS. Methylene blue for distributive shock: a potential new use of an old antidote. J Med Toxicol. (2013) 9:242. 10.1007/s13181-013-0298-7 23580172 PMC3770994

[10] Donati A Conti G Loggi S Preiser JC. Does methylene blue administration to septic shock patients affect vascular permeability and blood volume? Crit Care Med. (2002) 30:2271–7. 10.1097/00003246-200210000-00015 12394955

[11] Weingartner R Oliveira E Oliveira ES Friedman G. Blockade of the action of nitric oxide in human septic shock increases systemic vascular resistance and has detrimental effects on pulmonary function after a short infusion of methylene blue. Brazil J Med Biol Res. (1999) 32:1505–13. 10.1590/S0100-879X1999001200009 10585632

[12] Kirov MY Evgenov OV Evgenov NV Egorina EM Sovershaev MA Sveinbjørnsson B . Infusion of methylene blue in human septic shock: a pilot, randomized, controlled study. Crit Care Med. (2001) 29:1860–7. 10.1097/00003246-200110000-00002 11588440

[13] Dumbarton TC Minor S Yeung CK Green R. Prolonged methylene blue infusion in refractory septic shock: a case report. Canad J Anesth. (2011) 58:401–5. 10.1007/s12630-011-9458-x 21246318

[14] Brown G Frankl D Phang T. Continuous infusion of methylene blue for septic shock. Postgrad Med J. (1996) 72:612–4. 10.1136/pgmj.72.852.612 8977944 PMC2398584

[15] Porizka M Kopecky P Dvorakova H Kunstyr J Balik M. Methylene blue administration in patients with refractory distributive shock-a retrospective study. Sci Rep-UK. (2020) 10:1–8. 10.1038/s41598-020-58828-4 32020043 PMC7000741

[16] Li QS. Application of methylene blue in septic shock. Diet Health. (2021) 9:79.

[17] Kram SJ Kram BL Cook JC Ohman KL Ghadimi K. Hydroxocobalamin or methylene blue for vasoplegic syndrome in adult cardiothoracic surgery. J Cardiothor Vasc Ann. (2021) 36:469–76. 10.1053/j.jvca.2021.05.042 34176677

[18] Furnish C Mueller SW Kiser TH Beyer JT. Hydroxocobalamin versus methylene blue for vasoplegic syndrome in cardiothoracic surgery: a retrospective cohort. J Cardiothor Vasc Ann. (2020) 34:1763–70. 10.1053/j.jvca.2020.01.033 32115360

[19] Kavanaugh M Berumen J Chu F Yin J Beitler J. Methylene blue utilization for refractory septic shock in the setting of cirrhosis as a bridge to successful liver-kidney transplant: case report and review of the literature. Chest. (2015) 148:206. 10.1378/chest.2267413

[20] Evora PRB. Methylene blue does not have to be considered only as rescue therapy for distributive shock. J Med Toxicol. (2013) 9:426–426. 10.1007/s13181-013-0333-8 24078299 PMC3846965

[21] Moher D Liberati A Tetzlaff J Altman DG PRISMA Group^*^. Preferred reporting items for systematic reviews and meta-analyses: the PRISMA statement. PLoS Med. (2009) 6:e1000097. 10.1371/journal.pmed.1000097 19621072 PMC2707599

[22] Higgins PTJ Deeks J Altman D. Cochrane Handbook: Special Topics. Ch 16: Special topics in statistics. (2011) p. 389–432.

[23] Wells G. The Newcastle-Ottawa Scale (NOS) for assessing the quality of nonrandomized studies in meta-analysis. In: Symposium on Systematic Reviews: Beyond the Basics. (2014).

[24] Memis D Karamanlioglu B Yuksel M Gemlik I Pamukcu Z. The influence of methylene blue infusion on cytokine levels during severe sepsis. Anaesth Intens Care. (2002) 30:755–62. 10.1177/0310057X0203000606 12500513

[25] Habib AM Elsherbeny AG Almehizia RA. Methylene blue for vasoplegic syndrome postcardiac surgery. Indian J Crit Care Med. (2018) 22:168–73. 10.4103/ijccm.IJCCM_494_17 29657374 PMC5879859

[26] Saha A Jennings DL Ning Y Kurlansky P Miltiades AN Spellman JL . Methylene blue does not improve vasoplegia after left ventricular assist device implantation. Ann Thorac Surg. (2020) 111:800–8. 10.1016/j.athoracsur.2020.05.172 32758558

[27] Xiong XQ Jin LD Wang LR Zhu TQ Peng Y Lin LN. Effect of methylene blue on oxygen metabolism in patients wIm septic shock. China J Anesthesiol. (2010) 30:4.

[28] Zhang Z Wu XD. Therapeutic effect of methylene blue on septic shock. Contin Med Educ. (2016) 30:3.

[29] Lu YP Yu HJ Liu QY Yao M Zhu JG. Efficacy of continuous intravenous infusion of methylene blue in patients with septic shock. Nat Med J China. (2019) 99:4.

[30] Zhang JM Chen GF Chen NY Cheng GD. Efficacy analysis of methylene blue and norepinephrine in the treatment of va- soparalysis syndrome after cardiac surgery. World J Complex Med. (2021) 7:4.

[31] Koelzow H Gedney JA Baumann J Bellamy MC. The effect of methylene blue on the hemodynamic changes during ischemia reperfusion injury in orthotopic liver transplantation. Anesth Analg. (2002) 94:824–9. 10.1097/00000539-200204000-00009 11916779

[32] Maslow AD Stearns G Batula P Schwartz CS Gough J Singh AK. The hemodynamic effects of methylene blue when administered at the onset of cardiopulmonary bypass. Anesth Analg. (2006) 103:2–8. 10.1213/01.ane.0000221261.25310.fe 16790616

[33] Levin RL Degrange MA Bruno GF Boullon FJ. Methylene blue reduces mortality and morbidity in vasoplegic patients after cardiac surgery. Ann Thorac Surg. (2004) 77:496–9. 10.1016/S0003-4975(03)01510-8 14759425

[34] Ma YF Wei LJ Feng Y Guo ZH. Effect of methylene blue in infective endocarditis patients undergoing cardiac valve replacement with cardiopulmonary bypass. J Clin Anesthesiol. (2019) 35:4.

[35] Kofler O Simbeck M Tomasi R Hinske LC Klotz LV Uhle F . Early Use of Methylene Blue in Vasoplegic Syndrome: A 10-Year Propensity Score-Matched Cohort Study. J Clin Med. (2022) 11:1121. 10.3390/jcm11041121 35207394 PMC8880443

[36] Li WM. Impact study of methylene blue on adult septic shock hemodynamics. China Mod Med. (2014) 21:3.9869542

[37] Perdhana F Kloping NA Witarto AP Nugraha D Rehatta NM. Methylene blue for vasoplegic syndrome in cardiopulmonary bypass surgery: A systematic review and meta-analysis. Asian Cardiov Thoracic Ann. (2021) 29:717–28. 10.1177/0218492321998523 33653154

[38] Pasin L Umbrello M Greco T Landoni G. Methylene blue as a vasopressor: a meta-analysis of randomised trials. Critical Care Resuscit. (2013) 15:42–8. 23432501

[39] Evora PR Ribeiro PJ Vicente WV Reis CL Rodrigues AJ Menardi AC . Methylene blue for vasoplegic syndrome treatment in heart surgery. Fifteen years of questions, answers, doubts and certainties. Brazil J Cardiov Surg. (2009) 24:279–88. 10.1590/S0102-76382009000400005 20011872

[40] Juffermans NP Vervloet MG Daemen-Gubbels CR Groeneveld AB. A dose-finding study of methylene blue to inhibit nitric oxide actions in the hemodynamics of human septic shock. Nitric Oxide. (2010) 22:275–80. 10.1016/j.niox.2010.01.006 20109575

[41] Evans L Rhodes A Alhazzan W Antonelli M Coopersmith CM French C . Surviving sepsis campaign: international guidelines for management of sepsis and septic shock 2021. Intens Care Med. (2021) 47:1181–247. 10.1007/s00134-021-06506-y 34599691 PMC8486643

[42] Backer DD Foulon P. Minimizing catecholamines and optimizing perfusion. Crit Care. (2019) 23:149. 10.1186/s13054-019-2433-6 31200777 PMC6570631

[43] Mehaffey JH Johnston LE Hawkins RB Charles EJ Yarboro L Kern JA . Methylene blue for vasoplegic syndrome after cardiac operation: early administration improves survival. Ann Thorac Surg. (2017) 104:36–41. 10.1016/j.athoracsur.2017.02.057 28551045 PMC5523819

[44] Mccartney SL Duce L Ghadimi K. Intraoperative vasoplegia: methylene blue to the rescue! Curr Opin Anaesthesiol. (2018) 31:43–9. 10.1097/ACO.0000000000000548 29176374

[45] Wieruszewski PM Khanna AK. Vasopressor choice and timing in vasodilatory shock. Crit Care. (2022) 26:76. 10.1186/s13054-022-03911-7 35337346 PMC8957156

[46] Zhang XF Yun G Pan PF Wang Y Li WZ Yu XY. Methylene blue in the treatment of vasodilatory shock: a meta-analysis. Chin Crit Care Med. (2017) 29:982–7. 29151412 10.3760/cma.j.issn.2095-4352.2017.11.005

[47] Zhang CC Niu F Wu L Zhang CL Hao C Ma AY . Correlation of arterial blood lactic acid level in patients with septic shock and mortality 28 days after entering the intensive care unit. J Chin Physic. (2021) 23:5.

[48] Arevalo VN Bullerwell ML. Methylene blue as an adjunct to treat vasoplegia in patients undergoing cardiac surgery requiring cardiopulmonary bypass: a literature review. AANA J. (2018) 86:455–63. 31584419

[49] Martino E Winterton D Nardelli P Pasin L Calabrò M Bove T . The blue coma: the role of methylene blue in unexplained coma after cardiac surgery: a case series. J Cardiothorac Vasc Anesth. (2016) 30:423–7. 10.1053/j.jvca.2015.09.011 26703972

[50] Lv WY Tang WX Wei PH Li JJ. Use of methylene blue in vasoplegic syndrome. Int J Anesthesiol Resuscit. (2020) 41:5.

